# Gitelman syndrome with diabetes and kidney stones: A case report

**DOI:** 10.1097/MD.0000000000041207

**Published:** 2025-01-10

**Authors:** Huishan Wu, Xiongwei Ye, Meng Li

**Affiliations:** a The Second School of Clinical Medicine, Zhejiang Chinese Medical University, Hangzhou, China; b The Department of Clinical Laboratory, Zhejiang Hospital, Hangzhou, China.

**Keywords:** diabetes mellitus, Gitelman syndrome, hydrochlorothiazide test, kidney stones, single heterozygous mutation

## Abstract

**Rationale:**

Gitelman syndrome (GS) is a rare hereditary electrolyte disorder caused by mutations in the SLC12A3 gene. There is limited literature on the role of hydrochlorothiazide (HCT) testing and the SLC12A3 single heterozygous mutation in the diagnosis and management of patients with GS. In addition, cases of GS with concomitant kidney stones are rare.

**Patient concerns:**

A 48-year-old male patient suffered from unexplained hypokalemia for >10 years.

**Diagnoses:**

The patient was diagnosed with GS, type 2 diabetes mellitus, and kidney stones.

**Interventions:**

He was given potassium chloride sustained-release tablets and potassium magnesium aspartate tablets.

**Outcomes:**

His irregular potassium supplementation and hypoglycemic therapy resulted in poor control of potassium and blood glucose levels.

**Lessons:**

When unexplained hypokalemia is observed, the HCT test can help with the diagnosis of GS. When genetic testing reveals that a patient only carries only 1 SLC12A3 mutant allele, he requires further genetic evaluation. The patient’s combination of kidney stones and cysts could not exclude the diagnosis of GS. Patients with GS and diabetes should be monitored for the development of diabetic ketoacidosis.

## 1. Introduction

Gitelman syndrome (GS) is a rare hereditary electrolyte disorder caused by mutations in the SLC12A3 gene, which encodes the thiazide-sensitive sodium-chloride cotransporter (NCC) in the distal convoluted tubule. GS is predominantly observed in adolescents, and its typical clinical manifestations include hypokalemia, metabolic alkalosis, hypomagnesemia, and activation of the renin-angiotensin-aldosterone system (RAAS).^[1]^ We present a case of GS with kidney stones, cysts, and diabetes mellitus.

## 2. Case report

A 48-year-old male patient was admitted to the Endocrinology Department of our hospital with symptoms of dizziness and fatigue. Laboratory tests revealed hypokalemia (serum potassium 2.34 mmol/L). The patient was diagnosed with hypokalemia after a colonoscopy more than ten years ago, without significant symptoms at that time. Despite potassium supplementation, serum potassium levels did not normalize. Since then, the patient has had recurrent episodes of hypokalemia of unknown etiology. Three years ago, the patient began experiencing intermittent fatigue that increased in frequency after sweating and resolved with intravenous and oral potassium supplementation. The patient takes potassium chloride tablets irregularly as needed, with serum potassium levels fluctuating between 2.01 and 2.93 mmol/L. He had a history of type 2 diabetes mellitus (T2DM) for more than a year without regular medication or blood glucose monitoring.

On admission, the patient’s vital signs were as follows: temperature 37.0°C, pulse 79 beats per minute, respiratory rate 18 breaths per minute, blood pressure 113/69 mm Hg, weight 61.5 kg, height 170 cm, and body mass index 21.28 kg/m². The cardiopulmonary and abdominal examinations were unremarkable. The muscular strength of the extremities was grade V. There was no swelling in the lower extremities.

**Figure 1.**
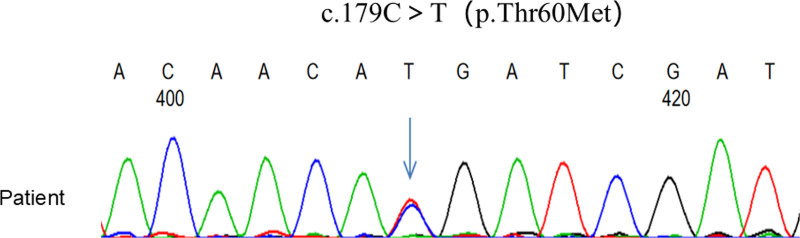
Genetic analysis of the SLC12A3 gene.

The patient’s laboratory data are summarized in Table 1. Serum biochemistry demonstrated hypokalemia (2.34 mmol/L) and hypomagnesemia (0.57 mmol/L). A 24-hour urinalysis revealed renal potassium loss (a K+/creatinine ratio of 11.9 mmol/mmol of creatinine, higher than 2 mmol/mmol of creatinine). The spot urine test revealed hypocalciuria (calcium-creatinine ratio of 0.07 mmol/mmol of creatinine, <0.2 mmol/mmol). Arterial blood gas analysis revealed the existence of compensatory metabolic alkalosis. Serum renin and aldosterone assays (standing) showed increased renin levels (291.73 mU/L). Adrenotropic hormone and cortisol rhythms were normal. Fasting blood glucose was 7.67 mmol/L, and glycosylated hemoglobin A1c was 6.5%. A urologic CT scan revealed bilateral kidney stones and cysts. Electrocardiography revealed sinus rhythm, 1 degree of atrioventricular block, and a considerable rightward shift in the electrical axis. The thyroid ultrasound and pituitary MRI showed no significant abnormalities. The hydrochlorothiazide (HCT) test was performed on the patient, which showed the net and relative increase in the fraction of chloride excretion (FECl, the net and relative increase measured as ΔFECl) of 0.81% and 36.5%, respectively (Table 2). With the consent of the patient and his family, we collected peripheral blood from the patient and his son for whole-exome sequencing. The results showed that the patient carried a single heterozygous mutation in the SLC12A3 gene, namely a c.179C > T (p.Thr60Met) mutation. A base 179 mutation from cytosine to thymine in exon 1, located in band 3 of region 1 of the long arm of chromosome 16, resulted in a substitution from threonine to methionine at amino acid 60 of the protein it encodes (Fig. 1).

**Table 1:** Laboratory examinations.

Test items	Specific items	Measured values	Normal values
Diabetes related tests	Fasting glucose (mol/L)	7.67↑	3.90–6.10
	Glycosylated hemoglobin (%)	6.5	4.0–6.0
Blood gas analysis	Lactate (mmol/L)		
	pH	7.449	7.35–7.45
	PaCO_2_ (mm Hg)	45.1↑	35.0–45.0
	cBase (ecf)	5.6↑	−3 to 3
	HCO_3_^−^ (mmol/L)	28.9↑	21.3–24.8
RAAS (standing)	Renin (mU/L)	291.73↑	4.7–47.6
	Aldosterone (ng/dL)	27.46	3.1–35.1
	Aldosterone/renin concentration ratio	0.09	
Adrenocorticotropic Hormone and cortisol tests	Cor (nmol/L, 8 am)	517.17	176.58–629.05
	Cor (nmol/L, 4 pm)	144.38	0–275.9
	ACTH (pg/mL, 8 am)	102.53↑	7–65
	ACTH (pg/mL, 4 pm)	40.62	
24-h urine electrolytes tests	Potassium (mmol/24 h)	100.50↑	25–100
	Calcium (mmol/24 h)	0.37	0–7.5
	Sodium (mmol/24 h)	385.84↑	130–260
	Phosphorus (mmol/L)	13.77	32.3–38.4
	Urine output (L/24 h)	2.2	
Kidney function tests	Creatinine (µmol/L)	77	59–104
	Urea (mmol/L)	5.5	2.8–7.2
	GFR (mL/min)	102.2	75–150

ACTH = adreno-cortico-tropic-hormone, Cor = cortisone, GFR = glomerular filtration rate, RAAS = renin-angiotensin-aldosterone system.

**Table 2:** Data of the HCT test.

Time (h)	Urine chloride (mmol/L)	Urine creatinine (µmol/L)	FECl (%)
0.5	74.24	2320	2.57
1	85.08	3670	1.86
1.5	88.60	2750	2.81
2	59.66	1890	2.75
2.5	33.54	2200	1.32
3	40.05	1400	2.42
3.5	58.75	1690	3.03
4	35.93	1090	2.88

ΔFECl = change in fraction of chloride excretion, HCT = hydrochlorothiazide.

FECl baseline mean (0.5 and 1 h) was 2.22%, maximal FECl after dosing was 3.03%, ΔFECl absolute value was 0.81% < 2.86%, ΔFECl relative value (increase from baseline) was 36.5% < 223%.

He was treated with potassium chloride sustained-release tablets and potassium magnesium aspartate tablets. Because of his irregular potassium supplementation and hypoglycemic therapy, both potassium and glucose were poorly controlled. A 1-and-a-half-year follow-up examination revealed that the patient’s fasting blood glucose level was 18 mmol/L, and his blood ketone bodies were 1.1 mmol/L.

## 3. Discussion

### 3.1. HCT test for the diagnosis of GS

The prevalence of GS in Europe is approximately 1/40,000.^[2]^ The exact prevalence in Asia is unknown, and the prevalence in Japan is 2/1000,^[3]^ which is higher than the European prevalence. The clinical diagnosis of GS relies on laboratory findings, which include hypokalemia, hypomagnesemia, hypocalciuria, and activation of the RAAS. Another inherited disorder with clinical manifestations similar to GS is Bartter syndrome (BS), which is caused by genetic mutations affecting the sodium-potassium-chloride cotransporter 2 and its associated regulatory proteins in the thick ascending limb of Henle’s loop. Patients with BS typically do not present with hypomagnesemia or hypocalciuria.^[4]^ Recent research has identified cases of GS with normal serum magnesium and urinary calcium levels,^[5]^ suggesting that differentiating GS from BS based solely on hypomagnesemia and hypocalciuria may lack specificity. To enhance the diagnostic accuracy of GS and BS, the clinical use of HCT and furosemide as diagnostic aids has been adopted. HCT exerts its effect by inhibiting the function of the NCC, while furosemide targets the sodium-potassium-chloride cotransporter 2, and both play an important role in identifying and diagnosing the site of renal tubular injury.^[6]^ Normally, there is a significant increase in FECl after the administration of hydrochlorothiazide. However, in patients with GS, due to the dysfunction of the NCC, the response to the HCT test is reduced, resulting in an increase in FECl that is substantially lower than the normal range. Peng et al determined the cutoff value of the HCT test for the diagnosis of GS (net ΔFECl ≤ 2.86% or relative ΔFECl ≤ 223%). The test has demonstrated sensitivities and specificities exceeding 95% in the diagnosis of GS, with a coincidence rate of 97.9% compared to genetic diagnosis.^[7]^ The accuracy and reliability of the HCT test in the diagnosis of GS was further validated. This patient’s net and relative ΔFECl were 0.81% and 36.5%, respectively, below the relevant cutoff value, confirming the GS clinical diagnosis.

However, there are several limitations to the use of the HCT test in clinical practice. Particularly in the pediatric patient population, there may be practical difficulties in collecting multiple urine specimens. In addition, the use of other medications that may affect electrolyte balance, such as other classes of diuretics or potassium supplements, may also interfere with the results of the HCT test. The ΔFECl values may be overestimated in patients with chronic renal insufficiency. Therefore, the use of the chloride and sodium clearance rates, rather than ΔFECl, to evaluate HCT test results is recommended.^[8]^

### 3.2. Genetic diagnosis of GS

In the diagnosis of GS, homozygous or compound heterozygous mutations in the SLC12A3 gene are crucial for definitive diagnosis, while single heterozygous mutations require a comprehensive evaluation combination with clinical symptoms. In this case, genetic testing of the patient revealed a heterozygous mutation c.179C > T (p.Thr60Met), which is one of the common mutation sites among Chinese GS patients.^[9]^ The clinical diagnosis of GS was confirmed integrating the patient’s clinical manifestations with the results of the HCT test. Despite the growing number of pathogenic SLC12A3 mutations found in patients with GS, it has been shown that up to 40% of patients still carry only 1 mutated SLC12A3 gene.^[10]^ In these patients with a single heterozygous mutation, there may be an undetected second mutant allele. This detection limitation may be related to several factors. Firstly, whole-exome sequencing can only detect mutations in exon coding regions and intron-exon junctions and cannot detect noncoding regions such as introns and large fragment rearrangements. Secondly, mutations may also be present in gene regulatory fragments such as promoter or enhancer fragments. Furthermore, it has been proposed that concurrent mutations may be present in genes other than the SLC12A3 gene, such as the CLCNKB gene, which is associated with Bartter syndrome type III.^[11]^ A study of 448 patients with a clinical diagnosis of GS for genetic mutations revealed that 18% of patients carried only 1 mutant allele by direct sequencing. A Multiplex ligation-dependent probe amplification assay and short fluorescent fragment quantitative multiplex PCR rescreening were used, and it was discovered that 6% of patients had a large rearrangement of the gene.^[12]^ This suggests that further development of genetic testing technologies is needed to identify all possible mutated loci.

There is debate regarding whether carriers of heterozygous mutations in the SLC12A3 gene exhibit a phenotype intermediate between GS patients and noncarriers. Previous studies have shown that heterozygous carriers have a milder clinical phenotype than compound heterozygotes and pure heterozygotes.^[13,14]^ However, following an expansion of the sample size of the study (147 heterozygous carriers, 5663 noncarriers), Wan et al^[15]^ discovered that heterozygous carriers had significantly lower serum potassium levels than noncarriers. The severe hypokalemia found in the single heterozygous patient in the present case is consistent with the findings of our study, which also suggests that the genetic heterogeneity of GS may be more complex than previously recognized. Unfortunately, the patient’s parents refused genetic testing for personal reasons, leaving the source of the patient’s mutation unclear.

### 3.3. GS combined with kidney stones and diabetes

According to the expert consensus on GS published by the Kidney Disease Improving Global Prognosis Organization (KDIGO) in 2017, kidney stones and kidney cysts are usually considered unfavorable features for the diagnosis of GS.^[2]^ However, Chen et al reported a case of a patient with GS confirmed by SLC12A3 gene testing, who presented with multiple bilateral kidney stones and cysts. High oxalate levels and low citrate levels were found in a 24-hour urine test, suggesting that kidney stone formation may be associated with increased urinary oxalate excretion rate and decreased urinary citrate excretion rate.^[16]^ The patient in this case also suffered from kidney stones and kidney cysts, challenging the previous view that kidney stones and cysts exclude a diagnosis of GS. The formation of kidney stones in the patient in this case may be related to the patient’s coexisting diabetes mellitus. In China, approximately 14% of patients with type 2 diabetes mellitus have coexisting kidney stones, a rate twice as high as that in the general population.^[17]^ Insulin resistance is linked to impaired renal tubular ammonia secretion,^[18]^ which causes lower urine pH. Persistently low urine pH is a pivotal factor in the formation of uric acid kidney stones.^[19]^ Furthermore, compensatory hyperinsulinemia related to insulin resistance may increase urinary calcium excretion, raising the risk of kidney stones.^[20]^ Currently, cases of GS complicated by kidney stones and kidney cysts are relatively rare, and more research is needed to elucidate the underlying mechanisms.

In addition, the patient was diagnosed with diabetes mellitus. The co-morbidity of diabetes mellitus is a significant clinical issue that should not be overlooked in GS patients. A study on the genotype-phenotype correlation in 67 patients with GS revealed that up to 47.8% of the patients exhibited abnormal glucose metabolism, with 19.4% of them diagnosed with T2DM.^[13]^ The pathogenesis of diabetes in patients with GS is closely related to a variety of pathophysiological factors, particularly hypokalemia, hypomagnesemia, and the activation of the RAAS. Hypokalemia may disrupt insulin production by interfering with the membrane potential of pancreatic β-cells.^[21]^ Magnesium ions, as key intracellular cofactors, play a role in regulating the activity of numerous enzymes. Low serum magnesium levels have been demonstrated to diminish the activity of insulin receptor tyrosine kinase, which subsequently impacts insulin signaling.^[22]^ In addition, the activation of RAAS results in elevated plasma levels of aldosterone, which promotes renal potassium excretion and sodium conservation, thereby exacerbating electrolyte disturbances. This affects insulin sensitivity and plays a role in the development of diabetes.^[23,24]^

In this case, the patient had a history of T2DM for more than 1 year. Furthermore, he had poor adherence to treatment, failing to take hypoglycemic medications regularly and monitor blood glucose levels. A follow-up examination conducted 1 and a half years later revealed that the blood glucose level was as high as 18 mmol/L, and the blood ketone body was 1.1 mmol/L, indicating diabetic ketosis. If not corrected in time, diabetic ketosis can progress to diabetic ketoacidosis (DKA). In patients with GS, the presence of DKA may be more difficult to detect since the metabolic alkalosis typical in GS patients may offset the metabolic acidosis of DKA, resulting in a seemingly normal pH.^[25,26]^ In addition, clinicians must be especially cautious about insulin therapy in patients with GS combined with DKA. This is because insulin may promote the intracellular transfer of potassium, thereby exacerbating the preexisting hypokalemia in patients with GS. Therefore, the existence of GS may complicate the diagnosis and management of DKA, necessitating rigorous blood glucose control in patients to prevent the start and progression of DKA.

## 4. Conclusion

In clinical practice, when encountering patients with unexplained hypokalemia, the HCT test is an important diagnostic tool that helps distinguish between GS and BS. When genetic testing reveals a single heterozygous mutation in GS, further genetic evaluation is necessary. The multiplex ligation-dependent probe amplification assay technique can help screen for the possible presence of large fragment rearrangements. Even in the presence of kidney stones and cysts, the diagnosis of GS should not be ruled out. For patients with GS, if glucose metabolism is abnormal, one must be vigilant for the occurrence of DKA. Electrolyte disturbances in GS patients may mask the metabolic acidosis of DKA, leading to a delayed diagnosis.

## Acknowledgments

This study was supported by the Zhejiang Provincial basic public welfare research project (No. GC22H206765).

## Author contributions

**Writing – original draft:** Huishan Wu.

**Writing – review & editing:** Xiongwei Ye, Meng Li.
